# Viriforms—A New Category of Classifiable Virus-Derived Genetic Elements

**DOI:** 10.3390/biom13020289

**Published:** 2023-02-03

**Authors:** Jens H. Kuhn, Eugene V. Koonin

**Affiliations:** 1 Integrated Research Facility at Fort Detrick, Division of Clinical Research, National Institute of Allergy and Infectious Diseases, National Institutes of Health, Fort Detrick, Frederick, MD 21702, USA; 2 National Center for Biotechnology Information, National Library of Medicine, National Institutes of Health, Bethesda, MD 20894, USA

**Keywords:** bracovirus, classification, gene transfer agent, GTA, ichnovirus, ICTV, *Polydnaviridae*, polydnavirus, taxonomy, viriform

## Abstract

The International Committee on Taxonomy of Viruses (ICTV) recently accepted viriforms as a new polyphyletic category of classifiable virus-derived genetic elements, juxtaposed to the polyphyletic virus, viroid, and satellite nucleic acid categories. Viriforms are endogenized former viruses that have been exapted by their cellular hosts to fulfill functions important for the host’s life cycle. While morphologically resembling virions, particles made by viriforms do not package the viriform genomes but instead transport host genetic material. Known viriforms are highly diverse: members of family *Polydnaviriformidae* (former *Polydnaviridae*) have thus far been found exclusively in the genomes of braconid and ichneumonid parasitoid wasps, whereas the completely unrelated gene transfer agents (GTAs) are widely distributed among prokaryotes. In addition, recent discoveries likely extend viriforms to mammalian genomes. Here, we briefly outline the properties of these viriform groups and the first accepted and proposed ICTV frameworks for viriform classification.

## 1. Introduction

In 2021, Koonin et al. outlined a definition of the term “virus” and assigned all mobile genetic elements (MGEs) that fit that definition sensu stricto into the orthovirosphere, and virus-like MGEs that possess some but not all distinctive features of viruses into the perivirosphere [1]. In parallel, this virus definition was officially proposed and shortly thereafter accepted by the International Committee on Taxonomy of Viruses (ICTV) [2,3] and subsequently became incorporated into the International Code of Virus Classification and Nomenclature (ICVCN). Accordingly, viruses are:

“…a type of MGEs that encode at least one protein that is a major component of the virion encasing the nucleic acid of the respective MGE and therefore the gene encoding the major virion protein itself; or MGEs that are clearly demonstrable to be members of a line of evolutionary descent of such major virion protein-encoding entities” (ICVCN Rule 3.3) [4].

This definition was created on operational/practical, rather than philosophical grounds; tobacco mosaic virus, the first virus described [5,6], was considered the “perfect” virus for establishment of a definition. All other MGEs undisputedly considered “viruses” by the virology community were then assessed for conformity with the first draft definition, which was subsequently iteratively modified to cover as many of them as possible, while striving to disrupt the established framework of virology to the least possible extent [1]. Indeed, the disruption was minimal, as almost all then-ICTV-classified viruses were found to fit the definition, with the notable exception of the family *Polydnaviridae*; conversely, MGEs not considered viruses but classified by the ICTV into separate categories (viroids and satellite nucleic acids) also kept their statuses, as they did not fit the new virus definition. This result enabled the formal definition of viroids and satellite nucleic acids for the ICVCN:

“Viroids are defined operationally by the ICTV as a type of MGEs that are uncoated, small, circular, single-stranded RNAs that do not encode proteins and do not depend on viruses for transmission, and that replicate autonomously through an RNA-RNA rolling-circle mechanism mediated by host enzymes and, in some cases, by *cis*-acting hammerhead ribozymes; or MGEs that are derived from a viroid in the course of evolution” (ICVCN Rule 3.3);

“Satellite nucleic acids are defined operationally by the ICTV as a type of non-viroid MGEs, which are dependent on viruses for replication and transmission; or MGEs that are derived from such entities in the course of evolution. (ICVCN Rule 3.3)” [2,3,4].

Polydnavirids, however, could not be added to these two categories. Because they did not fit within any of the definitions, a novel ICTV classification category was deemed to be required.

## 2. *Polydnaviriformidae*

Family *Polydnaviridae*, including a single genus *Polydnavirus*, was established by the ICTV in 1984 for a group of virus-like entities related to Campoletis sonorensis virus (CsV) [7]. In 1990, genus *Polydnavirus* was split into genera *Bracovirus* and *Ichnovirus*, with CsV becoming the type ichnovirus [8]. CsV and its relatives have multi-segmented (“poly”) DNA (“dna”) genomes that are permanently endogenized into the genomes of braconid or ichneumonid parasitoid wasps (order Hymenoptera: suborder Apocrita: superfamily Ichneumonoidea). These entities produce enveloped virion-like particles that, in contrast to true viruses, do not contain their genomes but instead contain encapsidated host-derived circular DNAs that, in the aggregate, range in length from about 190 to more than 500 kb. Female parasitoid wasps co-inject the particles along with their eggs into their (typically lepidopteran) insect prey, in which the encapsulated host DNA serves as a template for the expression of host proteins that inhibit the prey’s immune response, thereby enabling egg and offspring development [9,10,11,12].

The polydnavirid genomes can only be inherited vertically, from parental host to offspring, and the injection of the virion-like particles cannot result in an infection due to the absence of viral nucleic acids. CsV and its relatives are therefore de facto not MGEs. However, polydnavirids are distinct from typical endogenous viral elements (EVEs), which are remnants of virus genomes incorporated into host genomes because they constitute a functional system that goes beyond the expression of individual proteins. Thus, “polydna” entities, in contrast to classic EVEs, do not belong in either the orthovirosphere or the perivirosphere [1,13]. Consequently, the term “viriforms” was introduced [1,2] and officially defined in 2021 in the ICVCN as:

“…a type of virus-derived MGEs that have been exapted by their organismal (cellular) hosts to fulfill functions important for the host life cycle; or MGEs that are derived from such entities in the course of evolution” (ICVCN Rule 3.3),

with the comment that

“…MGEs previously classified in the family *Polydnaviridae* are considered to be viriforms in classification and nomenclature” [4].

In 2021, a taxonomic proposal was submitted to the ICTV proposing the renaming of family *Polydnaviridae* (→*Polydnaviriformidae*), genera *Bracovirus* (→*Bracoviriform*) and *Ichnovirus* (→*Ichnoviriform*), and all included species and “viruses” (→viriforms) [14]. This proposal was accepted in 2022 [15]; the current official taxonomy of family *Polydnaviriformidae* is outlined in Table 1. (Note that Campoletis sonorensis virus (CsV), highlighted in bold in Table 1, has been renamed Campoletis sonorensis ichnoviriform [CsIVf], with “Vf” standing for “viriform”).

Unfortunately, the current polydnaviriformid taxonomy is grossly misleading for several reasons. Analyses of polydnaviriformid genomes indeed identified (often deteriorated) virus-derived genes and thus substantiated the view of viriforms being former viruses that were “domesticated” by their hosts [16]. However, these analyses also demonstrated the polyphyly of family *Polydnaviridae*. Bracoviriforms are highly likely derivatives of an ancient member of the betanudiviral clade of family *Nudiviridae* [11,12,16,17,18] which, together with families *Baculoviridae*, *Hytrosaviridae*, and *Nimaviridae*, constitute the double-stranded DNA virus class *Naldaviricetes* [19] that is currently unassigned but likely belongs in the realm *Varidnaviria* [20,21,22]. By contrast, there is no evidence of ichnoviriforms being related to bracoviriforms and/or deriving from nudivirids, and their possible virus ancestor remains elusive [11,12,16,17,18,23].

Both bracoviriforms and ichnoviriforms are likely massively undersampled. Hymenopteran superfamily Ichneumonoidea, which includes parasitoid wasp families Braconidae, Ichneumonidae, and Trachypetidae, has ≈48,000 validly described members, but the actual number is likely much higher. Bracoviriforms have thus far only been identified in chelonine and microgastrine braconids, whereas ichnoviriforms have only been identified in banchine and campoplegine ichneumoids. (For classified viriforms, see Table 1 [24].) Even if one assumes that numerous other braconid and ichneuminid subtaxa do not harbor viriforms, this would indicate potentially thousands to tens of thousands of viriforms to be added to Table 1. Similar to EVEs, the viriform diversity estimates are hampered by the question of whether distinct viriform loci in ichneumonoidean genomes, and the sequence divergence between these, is due to independent domestication of distinct viruses or to only a few ancestral domestication events with subsequent speciation of the domesticating host. In the former case, rapid expansion of Table 1 could be expected; in the latter case, though, Table 1 might be reducible to a small number of species. Indeed, a recent phylogenomic study suggests that viriforms entered ichneumonoidean genomes only a handful of times [24].

However, bracoviriforms and ichnoviriforms may not be the only viriforms, and they many not only be found in parasitoid wasps. For instance, several unrelated, apparently nudivirid-derived viriform-like entities have recently been described. They include a hemipteran EVE that does not seem to produce particles, coleopteran bracoviriforms, a bracoviriform-unrelated viriform-like EVE in a campoplegine ichneumonid that produces particles that package host proteins instead of host DNA, and a viriform-like entity in an opiniine braconid [11,16]. Together, these findings indicate that the entire taxonomy of ichnopneumonoid viriforms will have to be thoroughly revised, starting with the abolishment of the apparently polyphyletic family *Polydnaviriformide*. The currently known insect viriforms are likely to represent only the proverbial tip of the iceberg of the actual diversity.

## 3. Gene Transfer Agents

Certain endogenized virus-derived elements in prokaryotes, commonly referred to as gene transfer agents (GTAs), have lifecycles similar to those of bracoviriforms or ichnoviriforms, although are evolutionarily unrelated to the latter [10,13]. Particles produced by GTAs resemble those of duplodnavirian caudoviricetes (“tailed phages”), although they are smaller than typical caudoviricete particles. However, GTAs package mostly random pieces of host DNA (which on some occasions include some of the GTA genes themselves). Consequently, GTAs are not infectious and are vertically inherited through the prokaryotic host cell division process. The GTA-encoding genes are distributed across multiple loci of the host genome. Their expression and GTA production are induced under stress conditions, in particular during starvation. The burst of GTA production kills the host cell resulting in the release of numerous GTA particles that enter neighbor cells, primarily those of the same species, mediating horizontal gene transfer (HGT) and increasing survival chances of the population [25,26,27,28,29].

The first GTA, now known as Rhodobacter capsulatus gene transfer agent (RcGTA), was described in 1974 as an uncharacterized agent facilitating “genetic exchange” among *Rhodopseudomonas capsulata* [30] (today *Rhodobacter capsulatus* [phylum *Pseudomonadota*: class *Alphaproteobacteria*]).

ICVCN Rule 3.3., from the original proposal to add viriforms to the ICTV framework [2], anticipates classification of GTAs with the comment that,

“Gene transfer agents (GTAs)…are considered to be viriforms in classification and nomenclature” [4].

Subsequently, Kogay et al. outlined and officially proposed a taxonomic framework for GTAs in 2022 [31,32]. This proposal was approved by the ICTV Executive Committee but awaits the next annual ratification vote, which will occur in early 2023. The framework consists of three initial GTA-specific viriform families (“-*gtaviriformidae*”) for the thus-far best-characterized GTAs [30,33,34,35,36,37,38], including the foundational RcGTA (Table 2).

However, similar to the current taxonomy of bracoviriforms and ichnoviriforms, the new framework for GTAs can only be seen as a first step in the systematic classification of prokaryotic virifoms. First, although all GTAs listed in the table are derived from caudoviricete ancestors, the viriforms of the three proposed families are not directly related to each other [31]. Second, GTAs are likely undersampled. Numerous GTAs have been discovered since RcGTA was first described, not only in highly diverse alphaproteobacteria [28,39,40,41], including even endosymbionts of diplonemid protists [42,43] and bacteria of the phylum *Spirochaetota*, but also in bacteria of the phylum *Thermodesulfobacteriota* and even in euryarchaea [28,36,39,44,45,46]. Thus, in the future, numerous, highly diverse GTAs will probably need to be classified. Third, as in the case of the polydnaviriformids, GTA diversity estimates are hampered by the unanswered question of how many times prokaryotes domesticated viruses to form GTAs versus how many GTAs can be traced back to a single domestication event. Many domestications would vastly expand Table 2, which was constructed based the current understanding of the three proposed families being traceable to at least three independent caudoviricete ancestors [31]. Fourth, GTA-like viriforms could have evolved from members of duplodnavirian megataxa other than *Caudoviricetes*, such as the recently described candidate class “*Mirusviricota*” [47], and/or from members of the realm *Varidnaviricota* infecting prokaryotes (“tailless phages”).

## 4. Discussion

“Viriforms” is an umbrella term for a variety of unrelated virus-derived genetic elements that is envisioned to be used similarly to the terms “viruses”, “viroids”, and “satellite nucleic acids”. All of these terms denote a range of polyphyletic entities and hence do not imply common evolutionary origins but rather reflect particular “lifestyles” of their members and are therefore of practical use.

The GTA-type viriforms resemble, and superficially could be confused with, caudoviricete prophages (a form of EVE), and likewise, bracoviriforms resemble nudivirid EVEs. However, whereas prophages are dormant (latent) viruses that can be reactivated (unless and until they deteriorate, which is their common fate), GTA-type viriforms are complex virus-like systems that have, however, been fully domesticated. They do not incorporate full sets of genes encoding GTA components and therefore never complete an infectious cycle, thereby never producing progeny particles. Furthermore, GTAs evolved small particle heads that can only incorporate segments of host DNA about 5 kilobases in length, much shorter than a typical caudoviricete genome. Similarly, EVEs are only fragments of endogenized virus nucleic acids that may or may not be functional, whereas bracoviriforms and ichnoviriforms maintain most viral lifecycle features but do not incorporate (most of the) genes for the components of their particles that consequently remain non-infectious.

The similarity of EVEs and viriforms implies that many viriforms might have been overlooked. A particularly interesting question is whether there are viriforms in animals beyond hymenopteran insects. The genomes of many animals, in particular vertebrates including mammals, are replete with retrovirid EVEs [48]. Strikingly, two cases have been described in which such endogenous retrovirid-like elements produce capsid (Gag) proteins that form non-infectious virion-like particles that incorporate either their own or heterogeneous mRNA [49,50]. The first example is Arc1, a fruit-fly protein with homologs in mammals. Arc1, via exapted retrovirid capsid (Gag) domains, forms virion-like, capsid-like structures that bind Arc1-encoding mRNA for transfer from motor neurons to muscles in a process that is dependent on retrotransposon-like sequences in a 3′ untranslated region of the mRNA [49,51,52,53]. The second example is a long terminal repeat (LTR) retrotransposon homolog, PEG10, that preferentially binds and facilitates vesicular secretion of its own mRNA [50]. Thus, both Arc1 and PEG10 are virus-derived elements that form virion-like particles that bind/pack nucleic acid cargo and are released from the producer cell (although within the same producer organisms) to fulfill important functions for the host, without producing infectious MGEs. Furthermore, an increasing number of diverse non-retrovirid EVEs is being discovered [54], compatible with the possibility that viriforms might be widespread, if not ubiquitous.

## Figures and Tables

**Table 1:** 2022 taxonomy of family *Polydnaviriformidae* [14].

Genus	Species	Viriform	Viriform Name Abbreviation	Host Subfamily	Host Family
*Bracoviriform*	*Bracoviriform altitudinis*	Chelonus altitudinis bracoviriform	CalBVf	Cheloninae	Braconidae
	*Bracoviriform argentifrontis*	Ascogaster argentifrons bracoviriform	AaBVf		
	*Bracoviriform blackburni*	Chelonus blackburni bracoviriform	CbBVf		
	*Bracoviriform curvimaculati*	Chelonus nr. curvimaculatus bracoviriform	CcBVf		
	*Bracoviriform flavitestaceae*	Phanerotoma flavitestacea bracoviriform	PfBVf		
	*Bracoviriform inaniti*	Chelonus inanitus bracoviriform	CinaBVf		
	*Bracoviriform insularis*	Chelonus insularis bracoviriform	CinsBVf		
	*Bracoviriform quadridentatae*	Ascogaster quadridentata bracoviriform	AqBVf		
	*Bracoviriform texani*	Chelonus texanus bracoviriform	CtBVf		
	*Bracoviriform canadense*	Hypomicrogaster canadensis bracoviriform	HcBVf	Microgastrinae	
	*Bracoviriform congregatae*	Cotesia congregata bracoviriform	CcBVf		
	*Bracoviriform crassicornis*	Apanteles crassicornis bracoviriform	AcBVf		
	*Bracoviriform croceipedis*	Microplitis croceipes bracoviriform	McBVf		
	*Bracoviriform demolitoris*	Microplitis demolitor bracoviriform	MdBVf		
	*Bracoviriform ectdytolophae*	Hypomicrogaster ectdytolophae bracoviriform	HcEVf		
	*Bracoviriform facetosae*	Diolcogaster facetosa bracoviriform	DfBVf		
	*Bracoviriform flavicoxis*	Glyptapanteles flavicoxis bracoviriform	GflBVf		
	*Bracoviriform flavipedis*	Cotesia flavipes bracoviriform	CfBVf		
	*Bracoviriform fumiferanae*	Apanteles fumiferanae bracoviriform	AfBVf		
	*Bracoviriform glomeratae*	Cotesia glomerata bracoviriform	CgBVf		
	*Bracoviriform hyphantriae*	Cotesia hyphantriae bracoviriform	ChBVf		
	*Bracoviriform indiense*	Glyptapanteles indiensis bracoviriform	GiBVf		
	*Bracoviriform kariyai*	Cotesia kariyai bracoviriform	CkBVf		
	*Bracoviriform liparidis*	Glyptapanteles liparidis bracoviriform	GlBVf		
	*Bracoviriform marginiventris*	Cotesia marginiventris bracoviriform	CmaBVf		
	*Bracoviriform melanoscelae*	Cotesia melanoscela bracoviriform	CmeBVf		
	*Bracoviriform nigricipitis*	Cardiochiles nigriceps bracoviriform	CnBVf		
	*Bracoviriform ornigis*	Pholetesor ornigis bracoviriform	PoBVf		
	*Bracoviriform paleacritae*	Protapanteles paleacritae bracoviriform	PpBVf		
	*Bracoviriform rubeculae*	Cotesia rubecula bracoviriform	CrBVf		
	*Bracoviriform schaeferi*	Cotesia schaeferi bracoviriform	CsBVf		
	*Ichnoviriform fumiferanae*	Glypta fumiferanae ichnoviriform	GfIVf	Banchinae	Ichneumonidae
*Ichnoviriform*	*Ichnoviriform acronyctae*	Diadegma acronyctae ichnoviriform	DaIVf	Campopleginae	
	*Ichnoviriform annulipedis*	Hyposoter annulipes ichnoviriform	HaIVf		
	*Ichnoviriform aprilis*	Campoletis aprilis ichnoviriform	CaIVf		
	*Ichnoviriform arjunae*	Casinaria arjuna ichnoviriform	CarIVf		
	*Ichnoviriform benefactoris*	Olesicampe benefactor ichnoviriform	ObIVf		
	*Ichnoviriform eribori*	Eriborus terebrans ichnoviriform	EtIVf		
	*Ichnoviriform exiguae*	Hyposoter exiguae ichnoviriform	HeIVf		
	*Ichnoviriform flavicinctae*	Campoletis flavicincta ichnoviriform	CfIVf		
	*Ichnoviriform forcipatae*	Casinaria forcipata ichnoviriform	CfoIVf		
	*Ichnoviriform fugitivi*	Hyposoter fugitivus ichnoviriform	HfIVf		
	*Ichnoviriform geniculatae*	Olesicampe geniculatae ichnoviriform	OgIVf		
	*Ichnoviriform infestae*	Casinaria infesta ichnoviriform	CiIVf		
	*Ichnoviriform interrupti*	Diadegma interruptum ichnoviriform	DiIVf		
	*Ichnoviriform lymantriae*	Hyposoter lymantriae ichnoviriform	HlIVf		
	*Ichnoviriform montani*	Enytus montanus ichnoviriform	X-2Vf		
	*Ichnoviriform pilosuli*	Hyposoter pilosulus ichnoviriform	HpIVf		
	*Ichnoviriform rivalis*	Hyposoter rivalis ichnoviriform	HrIVf		
	*Ichnoviriform sonorense*	**Campoletis sonorensis ichnoviriform**	**CsIVf**		
	*Ichnoviriform tenuifemoris*	Synetaeris tenuifemur ichnoviriform	StIVf		
	*Ichnoviriform terebrantis*	Diadegma terebrans ichnoviriform	DtIVf		

Orange shading: viriforms found in braconid wasps (chelonine braconids and microgastrine braconids). Green shading: viriforms found in ichneumonid wasps (banchine ichneumonids and campoplegine ichneumonids).

**Table 2:** 2023 proposed taxonomy of gene transfer agents (GTAs) [31,32].

Family	Genus	Species	Viriform	Viriform Name Abbreviation	Host
*Bartogtaviriformidae*	*Bartonegtaviriform*	*Bartonegtaviriform* *andersoni*	Bartonella gene transfer agent	BaGTA	hyphomicrobial alphaproteobacteria
*Brachygtaviriformidae*	*Brachyspigtaviriform*	*Brachyspigtaviriform* *stantoni*	Brachyspira hyodysenteriae gene transfer agent	BhGTA	spirochaetotans
*Rhodogtaviriformidae*	*Dinogtaviriform*	*Dinogtaviriform* *tomaschi*	Dinoroseobacter shibae gene transfer agent	DsGTA	rhodobacteral alphaproteabacteria
	*Rhodobactegtaviriform*	*Rhodobactegtaviriform marrsi*	Rhodobacter capsulatus gene transfer agent	RcGTA	
	*Rhodovulugtaviriform*	*Rhodovulugtaviriform kikuchii*	Rhodovulum sulfidophilum gene transfer agent	RsGTA	
	*Ruegerigtaviriform*	*Ruegerigtaviriform cheni*	Ruegeria pomeroyi gene transfer agent	RpGTA	

Gold shading: viriforms found in alphaproteabacteria (rhodobacteral alphaproteabacteria and hyphomicrobial alphaproteobacteria). Pink shading: viriforms found in spirochaetotans.

## Data Availability

Not applicable.
